# Freiburg Neuropathology Case Conference: A 58-year-old Patient Presenting with Dizziness, Nausea and Diplopia 12 Months after Total Resection of a Left-sided Temporal Glioblastoma Multiforme

**DOI:** 10.1007/s00062-022-01224-4

**Published:** 2022-11-21

**Authors:** M. Schwabenland, U. Würtemberger, D. Cipriani, S. Timme-Bronsert, H. Füllgraf, M. Prinz, H. Urbach, D. Erny, C. A. Taschner

**Affiliations:** 1 Departments of Neuropathology, University of Freiburg, Freiburg, Germany; 2 Department of Neuroradiology, Medical Center—University of Freiburg, Breisacherstraße 64, 79106 Freiburg, Germany; 3 Neurosurgery, University of Freiburg, Freiburg, Germany; 4 Institute for Surgical Pathology, University of Freiburg, Freiburg, Germany; 5 Core Facility for Histopathology and Digital Pathology, University of Freiburg, Freiburg, Germany; 6 Faculty of Medicine, Medical Centre—University of Freiburg, Freiburg, Germany

**Keywords:** Primary dural lymphoma, Skull base meningeoma, Dural metastasis, Disseminating glioblastoma, Ecchordosis physaliphora, Solitary dural plasmocytoma

## Case Report

**Fig. 1:**
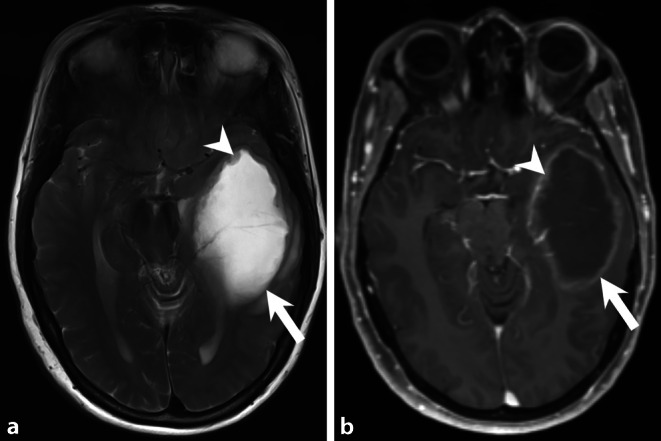
Initial preoperative imaging. Axial T2 weighted image (**a**) showed a space-occupying lesion of the left-sided temporal lobe (*arrow*). The center displayed major regressive changes with a slightly hyperintense tumor matrix at the lesion border (*arrowhead*). On axial T1 weighted image (**b**) after administration of gadolinium (Gd) the lesion showed a distinct circular enhancement (*arrow*) and enhancement of the tumor nodules (*arrowhead*)

**Fig. 2:**
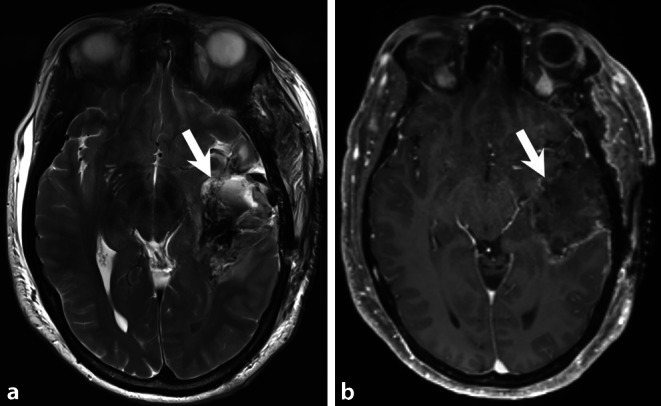
Initial postoperative imaging < 24 h after tumor removal. Axial T2 weighted image (**a**) showed a postoperative defect located in the left temporal lobe (*arrow*). Note the regressing midline shift. On axial T1 weighted image after administration of contrast (**b**) no Gd-enhancing residual tumor was present. The hyperintense structures along the operative defect (*arrow*) corresponded to hemosiderin

**Fig. 3:**
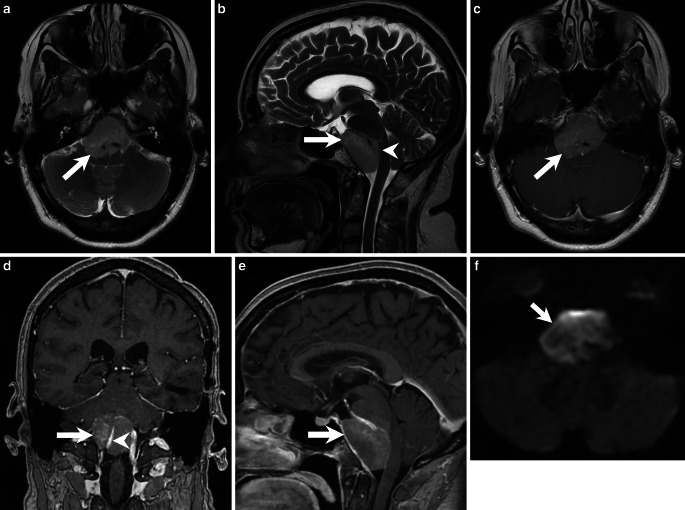
MR imaging 12 months after initial and total resection of the left temporal glioblastoma multiforme. Axial (**a**) and sagittal (**b**) T2 weighted images showed a space-occupying lesion (**a**,**b** *arrow*) located in the prepontine cistern. The lesion compressed and dislocated the brainstem and the myelon (*arrowhead*). On T1-weighted axial (**c**), coronal (**d**), and sagittal (**e**) MRI after administration of Gd the lesion showed some degree of homogeneous enhancement of contrast. The basilar artery was completely encased by the tumor (**d**, *arrowhead*). There was dural enhancement along the clivus. Thickening of the meninges as well as any direct relation to the clivus were absent. On diffusion-weighted images (b-value: 1000, **f**) the lesion (*arrow*) showed restricted diffusion

A 58-year-old female patient with a history of left temporal glioblastoma multiforme (GBM) World Health Organization (WHO) grade IV that had been diagnosed and treated by total tumor resection 12 months earlier (Figs. 1 and 2) presented to a peripheral hospital with a new onset of dizziness, nausea and diplopia. A magnetic resonance imaging (MRI) of the head was performed, which revealed a new space-occupying contrast-enhanced prepontine lesion (Fig. 3). At the left temporal resection site, no signs of a GBM recurrence were present. The patient was immediately transferred to our hospital. At the time of admission, the patient was awake and oriented with nausea and diplopia due to a palsy of the sixth cranial nerve. There were no clinical signs of swallowing difficulties or impairment of other cranial nerves. Due to the tumor location, the mass effect on the brainstem and the unknown etiology of the prepontine lesion, the indication for an open biopsy with reduction of the tumor was established. The day before surgery, the patient deteriorated neurologically and presented with respiratory insufficiency. A computed tomography (CT) of the head identified an obstructive hydrocephalus, which required an immediate placement of an external ventricular drainage.

Open biopsy and partial tumor resection were performed the following day with the patient under general anesthesia in a park bench position using intraoperative neurophysiological monitoring. The partial tumor resection was performed via a retrosigmoid approach. Macroscopically the tumor showed a grayish color, had a soft consistency and was adherent to the cranial nerves. Due to the adherence of the tumor in relation to the cranial nerves, a complete resection could not be performed.

The postoperative course on our intensive care unit showed missing swallowing and gag reflexes. An extubation could therefore not be performed, a tracheotomy was refused by the otherwise neurologically intact patient. In the following days an acute decrease of consciousness occurred. A CT of the head demonstrated an extensive tumor progression with compression of the brainstem. In fulfilment of the patient’s will, the treatment was discontinued and the patient passed away the following day.

## Imaging

Initial preoperative MRI shows a space-occupying lesion of the left-sided temporal lobe (Fig. 1, *arrows*). T2 weighted images (Fig. 1a, *arrow*) show regressive changes on T1 weighted images after administration of gadolinium (Gd). The lesion displays distinct contrast enhancement (Fig. 1b, *arrow* and *arrowhead*). Early postoperative (< 24 h) MRI obtained after tumor surgery confirms complete tumor resection (Fig. 2) but 12 months later the patient had MRI for dizziness, nausea, and diplopia (Fig. 3) showing a large space-occupying lesion (*arrow*) located in the prepontine cistern. The lesion compresses and dislocates the brainstem and the myelon (Fig. 3b, *arrowhead*). On T1-weighted MRI after administration of Gd the lesion shows some degree of homogeneous enhancement of contrast medium (Fig. 3c–e, arrow). On diffusion-weighted images (b-value: 1000), the lesion shows restricted diffusion (Fig. 3f, *arrow*).

## Differential Diagnosis

### Primary Dural Lymphoma

Primary central nervous system (CNS) lymphomas (PCNSL) account for a small proportion of all primary brain tumors and usually involve the brain parenchyma. A rare but distinct subtype of primary CNS lymphoma is primary dural lymphoma (PDL), which as a special feature histopathologically very often corresponds to low-grade B‑cell marginal zone lymphoma with a better prognosis, whereas most PCNSL are histopathologically diffuse large B‑cell lymphomas [1].

Primary dural lymphomas share similar imaging characteristics with other dural masses and can present as an extra-axial lobulated mass, which is hyperdense to brain parenchyma on non-enhanced CT imaging and especially in this modality cannot be distinguished with certainty from meningiomas. Bony involvement was described in about 3/4 of the cases in a case study [2].

In addition to the typical imaging features of extra-axial masses with broad-based dural attachment with a “dural tail” in contrast-enhanced sequences and cerebrospinal fluid rim (CSF cleft) in T2-weighted sequences, MRI shows strong and mostly homogeneous contrast enhancement as well as diffusion restriction with DWI hyperintensity and corresponding apparent diffusion coefficient (ADC) signal depression as signs of hypercellularity. In contrast to high-grade glioma, MR perfusion shows decreased relative cerebral blood volume (rCBV) values.

### Skull Base Meningioma

Skull base meningiomas are predominantly benign WHO grade 1 tumors when located at the skull base and only in rare cases WHO grade 2 or 3 tumors and then more often located along the convexity. On CT intralesional calcifications as well as hyperostotic changes of the adjacent bone can be seen. MRI shows the typical signs of an extra-axial, well-circumscribed tumor localization with the dural tail sign in contrast-enhanced sequences and the CSF cleft sign in T2/FLAIR (fluid attenuated inversion recovery) sequences. On MRI, calcification is best pictured with signal loss in SWI sequence. Contrast enhancement is usually homogeneous and strong, although meningiomas may also appear atypical with necrotic areas [3]. Depending on the location and size of the tumor a peritumoral edema is possible. Similarly, a direct space-occupying effect may occur due to, e.g. encasement of the vertebrobasilar arteries. In contrast to lymphoma, strong diffusion restriction is less typical.

### Dural Metastases

Dural metastases are among the most common causes of dural masses. The leading underlying primary tumors are breast, prostate, kidney, head and neck or lung cancer [4].

The cause may be direct extension of bony metastases, but also hematogenous, lymphathic, or via venous vertebral plexus retrograde metastases. Especially in the case of solitary occurrence of dural metastases, differentiation from other extra-axial masses such as meningioma or hemangiopericytoma is very often not reliably possible during initial imaging, as these entities may jointly show homogeneous contrast enhancement, calcifications and hyperostoses. Concomitant infiltration of the brain parenchyma or bony erosions in about 1/3 of cases each or infiltration of the cerebral sinuses is also described [4]. In addition to the typical signs of extra-axial location, advanced imaging techniques such as MR-spectroscopy can display an increased choline to creatine ratio as a marker for high cell turnover and an absence of the N-acetylaspartate (NAA) peak.

### Dural Metastasis of Glioblastoma

Glioblastoma multiforme (GBM) accounts for more than half of adult cases of malignant brain-derived tumors. MRI usually shows a typical appearance of an intra-axial mass with peripherally inhomogeneous, but strong contrast enhancement and cerebral blood volume increase on perfusion imaging, which usually surrounds a central zone of necrosis. In addition, peritumoral T2/FLAIR hyperintense edema is usually seen, with histologic evidence of tumor cell infiltration in addition to vasogenic changes. Most GBM patients experience tumor recurrence near the resection margins and despite infiltrative growth, metastases are generally rare and when they do occur are usually leptomeningeal extracranial via CSF seeding. Few cases of extraparenchymal dural metastases have been described worldwide in a subtype of glioblastoma with a primitive neuroectodermal component (PNET), although these may also occur after chemoradiotherapy [5].

### Ecchordosis Physaliphora and Intradural Clivus Chordoma

Ecchordosis physaliphora (EP) is a rare benign, in most cases retroclivally and prepontine located hamartomatous mass derived from notochord remnants, that is usually asymptomatic due to its size below 2 cm and should be differentiated from intradural clival chordoma, which also originates from ectopic notochord but is neoplastic [6].

In rare cases EP demonstrates space-occupying effects such as encasement of the basilar artery. CT shows a circumscribed bony substance defect, MRI usually shows a stalk-like connection to the clivus with predominantly T2-hyperintense, CSF isointense signal, and contrast enhancement is mostly absent. In contrast to this, intradural clivus chordoma demonstrates more frequently contrast enhancement after gadolinium injection.

### Solitary Dural Plasmocytoma

A rare differential diagnosis of dural space-occupying lesions is solitary dural plasmacytoma, which is a benign, single plasma cell tumor lesion without clinical and imaging-based evidence of systemic myeloma manifestation at the time of diagnosis but may evolve into malignant multiple myeloma later in life [7]. First choice of treatment is surgical resection followed by radiotherapy. There is a large imaging overlap with the here mentioned major differential diagnoses of dural bases masses, namely dural metastases and meningioma.

## Histology, Immunohistochemistry and Molecular Analyses

**Fig. 4:**
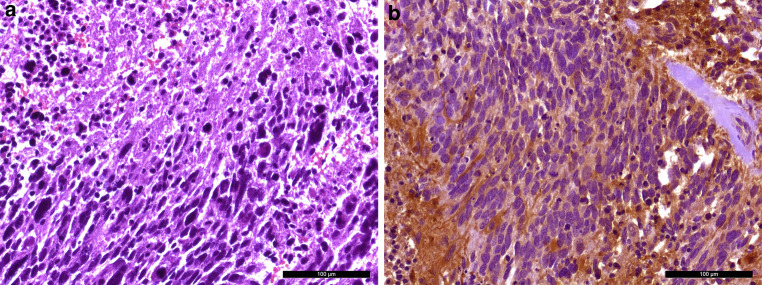
A hematoxylin and eosin (H&E) stained slide of the tumor (**a**) in the temporal cortex reveals a glial tumor with a diffuse infiltration pattern and necrotic areas. The tumor cells showed positive signal in the immunohistochemical reaction for glial fibrillary acidic protein (**b**, GFAP, *brown*). Hematoxylin (*blue*) was used as counterstaining. Scale bars: 100 µm

Nearly one year prior to the surgical removal of the clival tumor, we had assessed a tissue biopsy from the same patient from the temporal lobe. We identified a glial tumor with a diffuse infiltration into neighboring CNS tissue (Fig. 4a). The tumor cells looked pleomorphic and the nuclei appeared chromatin dense. Mitotic features and necrotic areas were visible. The tumor cells showed positivity in the immunohistochemical reaction for glial fibrillary acidic protein (GFAP, Fig. 4b). No R132H mutation of the IDH1 gene product was detectable immunohistochemically. After further immunohistochemical characterization, the patient was diagnosed with a glioblastoma, IDH-wildtype, CNS WHO grade 4.

**Fig. 5:**
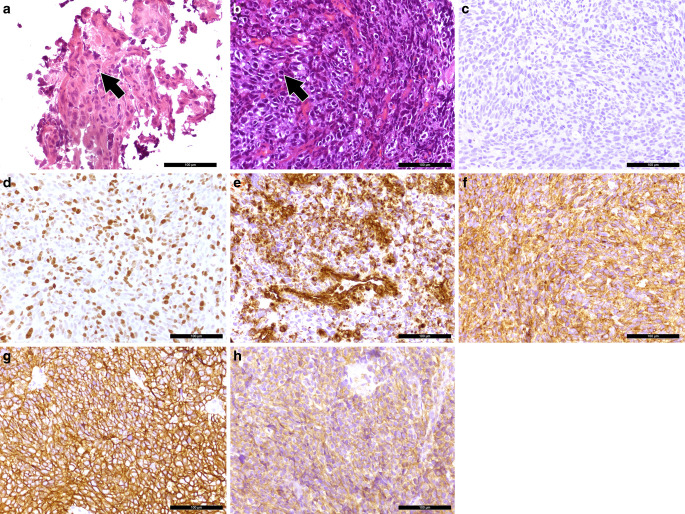
An intraoperative cryostat section stained with H&E (**a**) shows a pleomorphic tumor with mitotic features, as exemplarily indicated by an arrow. The H&E stained FFPE section (**b**) shows small fragments of a tumor with chromatin-dense nuclei and mitotic features (*arrow*). The immunohistochemical reaction for glial fibrillary acidic protein (GFAP, brown) remained negative (**c**). The tumor cells were highly proliferative, as indicated by the immunohistochemistry for Ki-67 (**d**, MIB‑1, *brown*). The immunohistochemistry for vimentin (**e**, *brown*) was partially positive. The tumor expressed synaptophysin (**f**, *brown*). Positivity was also observed in the immunohistochemical reaction for CD56 (**g**, *brown*). Tumor cells were also labelled in the immunohistochemical reaction for CD99 (**h**, *brown*). Hematoxylin (*blue*) was used as counterstaining for immunohistochemical reactions. Scale bars **a**–**h**: 100 µm

We now obtained a small tissue biopsy for intraoperative neuropathological assessment. A cryostat section was stained with hematoxylin and eosin (H&E, Fig. 5a). A pleomorphic tumor with a high cell density and mitotic features could be identified. Therefore, it was suspected that the tissue corresponds to the pre-existing glioblastoma. Additional tissue was fixed in formaldehyde and embedded in paraffin (FFPE).

An H&E stained FFPE tissue section showed connective tissue rich in collagen fibers as well as fresh bleedings. Several small fragments of the tumor could be observed (Fig. 5b). The nuclei were chromatin dense. Mitotic features were visible. Overall, the lesion resembled a small round blue cell tumor (SRBCT).

While the tumor cells of the pre-existing glioblastoma had stained positive for glial fibrillary acidic protein (GFAP), the immunohistochemical reaction for GFAP now remained negative (Fig. 5c). The tumor cells were also negative for oligodendrocyte transcription factor 2 (OLIG2). The nuclear expression of *ATRX* was preserved. An R132H mutation of the IDH1 gene product could not be detected immunohistochemically. The proliferation marker MIB‑1 labelled more than 60% of the tumor cells (Fig. 5d).

The immunohistochemical reactions for the pan-cytokeratin markers MNF116 and AE1/3 as well as epithelial membrane antigen (EMA), transcription termination factor 1 (TTF1) and melan‑A remained negative. Vascular structures were labelled in the immunohistochemical reaction for alpha-smooth muscle actin (SMA). Desmin, signal transducer and activator of transcription 6 (STAT6) and S100 were negative. Nuclear expression of integrase interactor 1 (INI-1) was preserved.

**Fig. 6:**
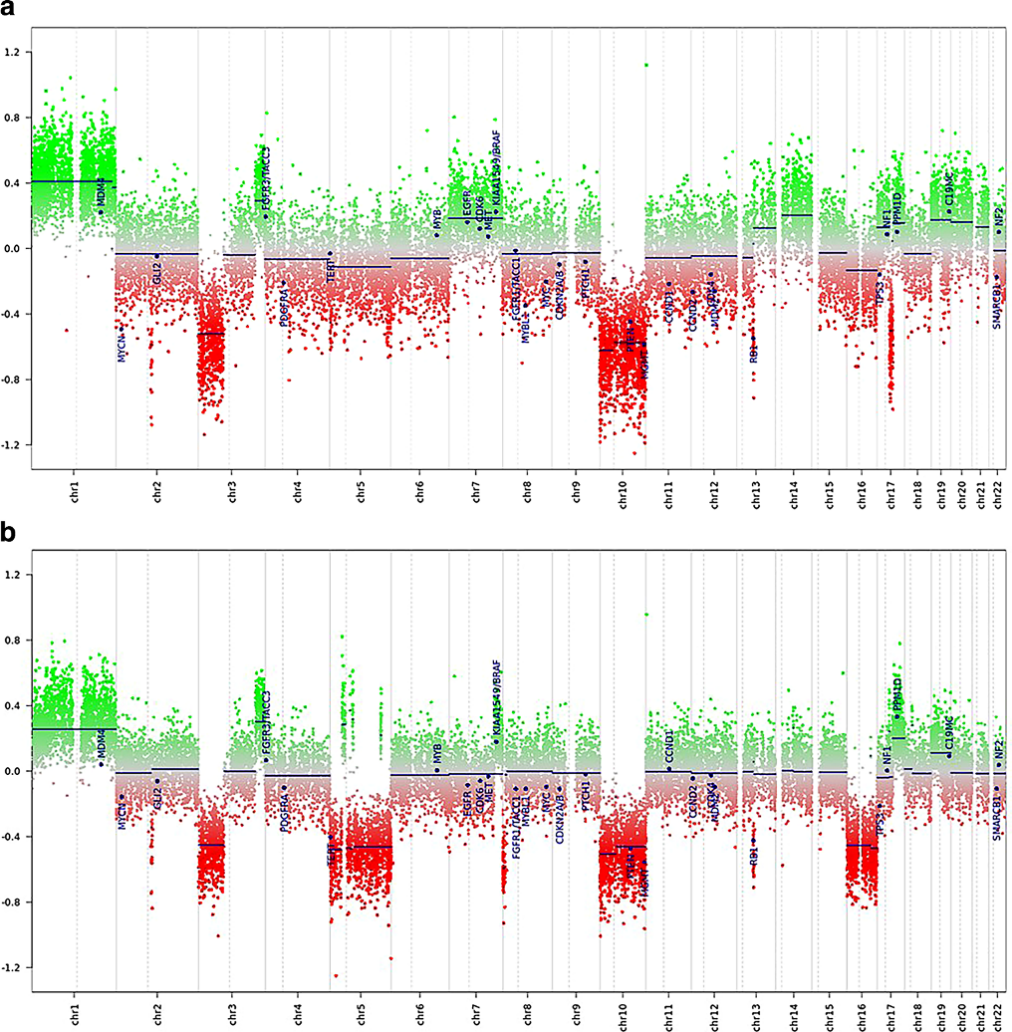
The copy number variation profiles of the tumor in the temporal lobe (**a**) and the clival tumor (**b**) are depicted

Only a few cells were labelled in the immunohistochemical reactions for leukocyte common antigen (LCA, CD45), B‑lymphocyte antigen CD20 and B‑cell antigen receptor complex-associated protein alpha chain (CD79a). The tumor cells showed partial positivity in the reaction for vimentin (Fig. 5e). Positivity was also observed in the reactions for synaptophysin (Fig. 5f) and neural cell adhesion molecule (NCAM, CD56, Fig. 5g), while chromogranin remained negative. The tumor cells also showed positive signal in the immunohistochemical reaction for CD99 (Fig. 5h). With the previous histological and immunohistochemical assessment, a clinically suspected lymphoma, chordoma and a chondrosarcoma could be excluded. The tumor’s immunohistochemical profile did not match the pre-existing glioblastoma. With synaptophysin and CD56 positivity, a metastasis of a neuroendocrine carcinoma was discussed. All additional immunohistochemical reactions (CDX2, CK19, CK20 and EP4) were negative. Since the tumor cells were expressing CD99, we have also discussed a Ewing sarcoma. No translocation involving the EWSR1 gene could be detected by chromogenic in situ hybridization (CISH). Next, an 850k DNA methylation assay of the current tumor as well as the tissue from the pre-existing glioblastoma was performed [8]. The methylation pattern was compared to a reference dataset, the brain tumor classifier, version 11b4. Both the previous tissue from the temporal lobe that has histologically and immunohistochemically been classified as a glioblastoma and the current biopsy showed the highest calibrated score for the methylation class family glioblastoma, IDH wildtype. The methylation pattern of both samples matched to the methylation class glioblastoma, IDH wildtype, subclass RTK II. We then applied the most recent reference dataset, the newly developed brain tumor classifier version 12.5. With a highest calibrated score of 0.9999 and 0.99964, both samples then matched to the methylation class glioblastoma, IDH-wildtype with primitive neuronal component (novel). The copy number variation profile showed the characteristics of glioblastomas with a primitive neuronal component (Fig. 6a,b). Both samples showed a loss of chromosome 10, which is observed in more than 90% of the glioblastomas with a primitive neuronal component [9]. Moreover, there was a gain in chromosome 1, which is observed in around half of the cases [9]. The copy number variation profile also showed alterations in RB1. No CDKN2A/B alterations were observed. The MGMT promotor was predicted to be methylation in both specimens. Taken together, the two tumor samples showed a different immunohistochemical pattern. The clival tumor showed no GFAP expression. The absence of GFAP, the high proliferation index and the CD99 positivity in particular led to the exploration of further potential differential diagnoses, e.g. a metastasis of a neuroendocrine carcinoma or a Ewing sarcoma. The result of the 850k methylation assay then revealed that both tumors share the same distinct methylation pattern. Therefore, the clival tumor has to be seen as a metastasis of the tumor in the temporal lobe. The methylation pattern of both tumors matched to the novel methylation class glioblastoma, IDH wildtype with primitive neuronal component. Given the loss of GFAP and the expression of synaptophysin and CD56 in the new clival biopsy, the tumor did certainly show the immunohistochemical features of a glioblastoma with a neuronal component. The immunohistochemical reactions for synaptophysin, CD56 and CD99 were subsequently also performed on the initial biopsy tissue from the temporal lobe. The tumor cells showed a strong positivity for synaptophysin and CD56. Furthermore, some tumor cell groups were weakly positivity for CD99. Despite moderate GFAP positivity, it is therefore reasonable to diagnose a glioblastoma with a neuronal component in the temporal lobe biopsy as well.

## Diagnosis

### Metastasis of a glioblastoma (WHO IV) with a primitive neuronal component

Malignant gliomas with a primitive neuronal component have first been described in 2009 [10]. Using an 850k methylation assay, a molecular subtype of glioblastomas that overlaps with the unique histological pattern of glioblastomas with a primitive neuronal component could recently be identified [9]. Of note, the tumor entity is extremely rare, since only 63 cases showed this distinct methylation profile when more than 75,000 tumor samples were compared [9]. The tumor cells typically show a loss of GFAP expression, but express neuronal markers such as synaptophysin or NSE [9]. A very high proliferation index is common [11]. Those tumors may therefore be misinterpreted as neuroendocrine carcinomas. Cerebrospinal fluid (CSF) and leptomeningeal dissemination seem to be common in this entity [9, 10, 12]. The copy number variation profile typically shows a loss of chromosome 10, a gain of chromosome 1 and alterations in RB1, TP53 and PTEN [9]. The median overall survival is 12 months [9].

The present case is highly interesting for several reasons. Firstly, glioblastomas with a primitive neuronal component are extremely rare. When more than 75,000 brain tumors were analyzed, only 63 harbored the same characteristic methylation profile that was detected in both of our specimens [9]. Secondly, those tumors were reported to have a high risk for cerebrospinal fluid and leptomeningeal dissemination. In the abovementioned cohort of 63 cases, clinical data were available for 10 patients. In four of them, leptomeningeal dissemination with spinal metastasis was reported [9]. In glioblastoma in general, leptomeningeal dissemination is reported to occur in around 4% of the patients [13]. With both the temporal and clival tumor sharing the same methylation pattern and matching to the novel methylation class glioblastoma, IDH wildtype with primitive neuronal component, it is reasonable to postulate that the clival tumor arose after leptomeningeal/cerebrospinal fluid dissemination from the tumor in the temporal lobe. Our present case therefore supports the previous findings that glioblastomas with primitive neuronal components show a higher risk of leptomeningeal and cerebrospinal fluid spread. Finally, the case is also interesting from a neuropathological point of view since the primary tumor did not present with a GFAP loss, but (already) had a distinct methylation pattern matching glioblastomas with a primitive neuronal component.
